# Association of the Glycemia Risk Index With Glycemic Metrics and Sensor Usage in a Real-World Pediatric Population With Low Hypoglycemia Rates in Saudi Arabia

**DOI:** 10.7759/cureus.90536

**Published:** 2025-08-19

**Authors:** Lamya M Alzubaidi, Faisal S El Enezi, Afaf I Alsagheir, Muna H Hassanein, Abdullah M Alsoheimi, Abdullah M Arabe, Amera S Alrshood, Abdulmohsen K Bakhsh

**Affiliations:** 1 Ada'a Health Center, Ministry of Health, Riyadh, SAU; 2 Assistant Deputyship of Hospitals’ Services, Therapeutic Deputyship, Ministry of Health, Riyadh, SAU; 3 Pediatric Endocrinology, King Faisal Specialist Hospital and Research Centre, Riyadh, SAU; 4 Training and Institutional Development, Ministry of Health, Riyadh, SAU; 5 General Directorate of Specialized Centers Affairs, Ministry of Health, Riyadh, SAU; 6 Kidney and Pancreas Transplant, Organ Transplant Center of Excellence, King Faisal Specialist Hospital and Research Centre, Riyadh, SAU

**Keywords:** continuous glucose monitoring, glycemia risk index, glycemic metrics, pediatrics, sensor usage time, type 1 diabetes mellitus

## Abstract

Introduction: The Glycemia Risk Index (GRI) is a recently developed composite measure designed to consolidate overall glycemic control into a single, interpretable score. The aim of this study was to investigate the associations between the GRI and glycemic metrics derived from continuous glucose monitoring (CGM), including sensor usage time, in children with diabetes using the FreeStyle Libre 2 Plus® CGM system (Abbott Diabetes Care, Witney, UK).

Method: De-identified CGM data from 147 pediatric patients with diabetes in Saudi Arabia, treated at two governmental hospitals between January 2023 and September 2024, were analyzed. Glycemic metrics from the ambulatory glucose profile were recorded, and the GRI and its hypoglycemia and hyperglycemia components were calculated. Correlations between the GRI, its components, and time in range with glycemic metrics were assessed using Pearson and Spearman correlation coefficients. The associations between GRI and its components and two sensor usage time groups (70%-89% versus >90%) were evaluated using the independent t-test and Mann-Whitney U test. A p-value < 0.05 was considered statistically significant.

Results: There was a negative correlation between GRI and time in range (r = -0.941), level one time above range (181-250 mg/dl; r = -0.447), time below range (< 70 mg/dl; r = -0.244), level one time below range (69-54 mg/dl; r = -0.248), and hypoglycemia component (r = -0.243) (all p-values <0.05). There was a positive correlation between GRI and average blood glucose (r = 0.923), glucose management indicator (r = 0.922), time above range (>180 mg/dl; r = 0.893), level two time above range (>250 mg/dl; r = 0.944), and hyperglycemia component (r = 0.929) (all p-values <0.05). Participants with lower sensor usage (70-89%) had significantly higher GRI values (median: 96.00; IQR: 75.80-100.00) compared to those with ≥90% usage (median: 82.40; IQR: 60.40-100.00; p = 0.004). The hyperglycemia component was also significantly higher in the lower usage group (mean: 53.65 vs. 46.62, p = 0.032).

Conclusion: Average glucose, glucose management indicator, and time in range showed negative correlations with GRI, while extreme hyperglycemia correlated positively. These results support GRI's role in assessing hyperglycemic exposure and treatment efficacy. Unexpected negative correlations with mild hyperglycemia and mild hypoglycemia warrant further studies. GRI and its hyperglycemic component improved with increased sensor usage, suggesting better glycemic control with higher CGM adherence.

## Introduction

The increasing incidence of type 1 diabetes mellitus (T1DM) among adolescents and young adults represents a growing public health concern worldwide. The Global Burden of Disease Study (2019) reported a rise from 7.78 to 11.07 patients per 100,000 population between 1990 and 2019 [1]. Saudi Arabia is among the countries with the highest incidence rates, with 109.5 cases per 100,000 reported in children and adolescents, surpassing rates observed in many high-income countries [2,3].

The use of continuous glucose monitoring (CGM) systems has increased substantially in recent years, particularly among pediatric and adolescent populations with T1DM, contributing to improved disease management [4]. These systems deliver real-time glucose data and trends, offering insights into glycemic patterns and control [5,6]. This is particularly advantageous for children who may have difficulty recognizing or communicating symptoms of hypo- or hyperglycemia [6].

Standardized metrics, visual tools like the ambulatory glucose profile (AGP), and defined targets have been developed to enhance the clinical utility of CGM data [5,7]. The international consensus on time in range (TIR) has established target thresholds for key CGM-derived metrics, including metrics of TIR, average blood glucose levels, and glycemic variability [5,7]. Assessing these metrics alongside AGP and hemoglobin A1c (HbA1c) helps clinicians evaluate glycemic control and guide personalized treatment adjustments [5,8].

Among CGM-derived metrics, TIR has emerged as a key measure of glycemic control, as it captures the day-to-day glycemic patterns experienced by individuals with diabetes [9]. Moreover, growing evidence indicates that TIR may also predict the long-term risk of diabetes-related complications [10]. However, due to the asymmetric distribution of out-of-range glucose values, TIR should be complemented by specific hypoglycemia indicators to adequately assess its frequency and severity [9].

To address the need for a metric that captures both hyperglycemia and hypoglycemia, the Glycemia Risk Index (GRI) was recently developed as a composite CGM-based measure of glycemic risk. It incorporates both hypoglycemic and hyperglycemic components, assigning greater weight to extreme values, and has shown stronger alignment with clinician assessments compared to other metrics like TIR [11].

Several studies have demonstrated the potential of the GRI as a valuable tool for assessing glycemic control and treatment safety in individuals with T1DM [8,12]. However, no data on the association of GRI with glycemic metrics in solely pediatric patients with T1DM have been reported in Saudi Arabia. The aim of the present study was to investigate the associations between the GRI and glycemic metrics derived from the CGM system, including CGM sensor usage time.

## Materials and methods

Sensors and readers

We conducted a secondary analysis of real-world CGM data from pediatric patients with T1DM. The data were originally collected as part of a larger study approved by the Institutional Review Board (IRB) of the Saudi Ministry of Health, targeting the association between glycemic metrics and the CGM system's daily scanning frequency. This current analysis focused on a subset of de-identified data from 147 children with T1DM who were receiving care at two government pediatric hospitals in Saudi Arabia. All patients were using the FreeStyle Libre 2® CGM system (Abbott Diabetes Care, Witney, UK). This system provides real-time interstitial glucose measurements and is designed for individuals aged four years and older. It measures glucose levels in interstitial fluid and is factory calibrated, eliminating the need for finger-stick calibration or manual coding. The system offers real-time glucose measurements every minute (1,440 readings daily) and supports a sensor lifespan of up to 14 days. When paired with personal computer-based software and an active internet connection, the reader’s 90-day memory is uploaded to a secure database. Data from this database, spanning from January 2023 to September 2024, were utilized for the current study. The reporting software requires a user agreement that authorizes the collection of de-identified data during each internet-connected session.

Glycemic measure

The following glycemic metrics were recorded: average blood glucose (ABG), time in range (TIR; 70-180 mg/dl), time below range (TBR; <70 mg/dl), level one time below range (TBR1; 54-69 mg/dl), level two time below range (TBR2; <54 mg/dl), time above range (TAR; >180 mg/dl), level one time above range (TAR1; 181-250 mg/dl), level two time above range (TAR2; >250 mg/dl), and the glucose management indicator (GMI).

We computed the hypoglycemic component (CHypo) of the GRI using the formula: $TBR2+(0.8\times TBR1)$. We computed the hyperglycemic component (CHyper) of the GRI using the formula: $TAR2+(0.5\times TAR1)$. The overall GRI was calculated using the following formula: $(3.0\times TBR2)+(2.4\times TBR1)+(1.6\times TAR2)+(0.8\times TAR1)$, with a maximum allowable value of 100 [11]. The "percentage of time the sensor is active” was utilized as a measure of CGM sensor usage. It represents the percentage of time the sensor successfully captured glucose data during its wear period.

Data analysis

Data analysis was performed using SPSS version 21.0 (IBM Corp., Armonk, NY). The Kolmogorov-Smirnov test was used to assess the normality of the variable distributions. Descriptive statistics are presented as mean and standard deviation (SD), and median and interquartile range (IQR). The correlation between GRI and each CGM metric was assessed using the Pearson correlation coefficient for normally distributed data and the Spearman correlation coefficient for non-normally distributed data. GRI was compared across two CGM sensor usage groups (70%-89% vs. <90%) using the independent t-test for normally distributed variables and the Mann-Whitney test for non-normally distributed variables. A GRI grid graph plotting CHypo on the horizontal axis and CHyper on the vertical axis was generated using the online tool provided by the Diabetes Technology Society at https://www.diabetestechnology.org/gri/. This tool offers a platform for calculating and visualizing glycemic risk data. Statistical significance was set at p < 0.05.

## Results

The dataset included 147 children with T1DM who were receiving insulin therapy. The glycemic metrics, including the ABG, GMI, TIR, TBR and its two levels (TBR1 and TBR2), TAR and its two levels (TAR1 and TAR2), GRI, CHypo, and CHyper are summarized in Table 1.

**Table 1:** The glycemic metrics, Glycemia Risk Index, hypoglycemia component, and hyperglycemia component (N = 147).

Glycemic metrics	Mean (standard deviation )
Average blood glucose	232.34 (56.16)
Glucose management indicator	8.87 (1.34)
Time in range	35.46 (17.22)
Time below range (<70 mg/dl)	2.88 (3.85)
Time below range level 1(69-54 mg/dl)	2.78 (3.50)
Time below range level 2(<54 mg/dl)	0.11 (.92)
Time above range (>180 mg/dl)	61.66 (18.80)
Time above range level 1 (181-250 mg/dl)	22.23 (6.86)
Time above range level 2 (>250 mg/dl)	39.43 (21.27)
Glycemia Risk Index	81.49 (21.47)
Hypoglycemia component	2.33 (3.18)
Hyperglycemia component	50.54 (19.78)

The results of the correlation between GRI and glycemic metrics are presented in Table 2. There was a strong negative correlation between GRI and TIR (r = -0.941, p < 0.001). There was a strong positive correlation between GRI and ABG (r = 0.923, p < 0.001), GMI (r = 0.922, p < 0.001), TAR (r = 0.893, p < 0.001), TAR2 (r = 0.944, p < 0.001), and CHyper (r = 0.929, p < 0.001). GRI had a moderate negative correlation with TAR1 (r = -0.447, p < 0.001) and weak negative correlations with TBR (r = -0.244, p = 0.003), TBR1 (r = -0.248, p = 0.002), and CHypo (r = -0.243, p = 0.003). The correlation with TBR2 was weak and not statistically significant (r = 0.039, p = 0.639).

**Table 2:** Correlation plot representing the relationships between Glycemia Risk Index and glycemic metrics (N = 147). GRI: Glycemia Risk Index; TIR: time in range (70-180 mg/dL); ABG: average blood glucose; GMI: glucose management indicator; TBR: time below range (<70 mg/dL); TBR1: time below range level one (69-54 mg/dL); TBR2: time below range level two (<54 mg/dL); TAR: time above range (>180 mg/dL); TAR1: time above range level one (181-250 mg/dL); TAR2: time above range level two (>250 mg/dL); CHypo: hypoglycemia component; CHyper: hyperglycemia component; R: correlation coefficient; P: p-value.

		GRI	TIR	ABG	GMI	TBR	TBR1	TBR2	TAR	TAR1	TAR2	CHypo	CHyper
GRI	R	1	-0.941	0.923	0.922	-0.244	-0.248	0.039	0.893	-0.447	0.942	-0.243	0.929
	P	-	<0.0010	<0.001	<0.001	0.003	0.002	0.639	<0.001	<0.001	<0.001	0.003	<0.001
TIR	R	0.941	1	-0.934	-0.933	0.423	0.425	0.066	-0.981	0.262	-0.978	0.422	-0.978
	P	<0.001	-	<0.001	<0.001	<0.001	<0.001	0.431	<0.001	0.001	<0.001	<0.001	<0.001

Similarly, TIR showed a strong negative correlation with ABG (r = -0.934, p < 0.001), GMI (r = -0.933, p < 0.001), TAR (r = -0.981, p < 0.001), TAR2 (r = -0.978, p < 0.001), and CHyper (r = -0.978, p < 0.001). TIR also showed moderate positive correlations with TBR (r = 0.423, p < 0.001), TBR1 (r = 0.425, p < 0.001), and CHypo (r = 0.422, p < 0.001), and showed a weak positive correlation with TAR1 (r = 0.262, p = 0.001). The correlation between TIR and TBR2 was very weak and not statistically significant (r = 0.066, p = 0.431).

Table 3 shows the glycemic risk metrics across two CGM sensor usage groups (70-89% vs. ≥90%). The group with lower sensor usage (70-89%) had significantly higher GRI values (median: 96.00; IQR: 75.80-100.00) compared to those with ≥90% usage (median: 82.40; IQR: 60.40-100.00; p = 0.004). CHyper was also significantly higher in the lower usage group (mean: 53.65 vs. 46.62, p = 0.032). No significant difference was observed in CHypo between the two groups (p = 0.113).

**Table 3:** Glycemia Risk Index, hypoglycemia component, and hyperglycemia component according to sensor usage time (N = 147). GRI: Glycemia Risk Index; CHypo: hypoglycemia component; CHyper: hyperglycemia component. P-values < 0.05 are considered statistically significant. Significant p-values are indicated with an asterisk (*).

	Sensor usage time		P-value
	70-89 (82)	90 and above (65)	
GRI	96.00 (75.80-100.00)	82.40 (60.40-100.00)	0.004*
CHypo	1.60 (0.80-3.20)	0.80 (0.00­-2.90)	0.113
CHyper	53.65 (18.70)	46.62 (20.53)	0.032*

**Figure 1:**
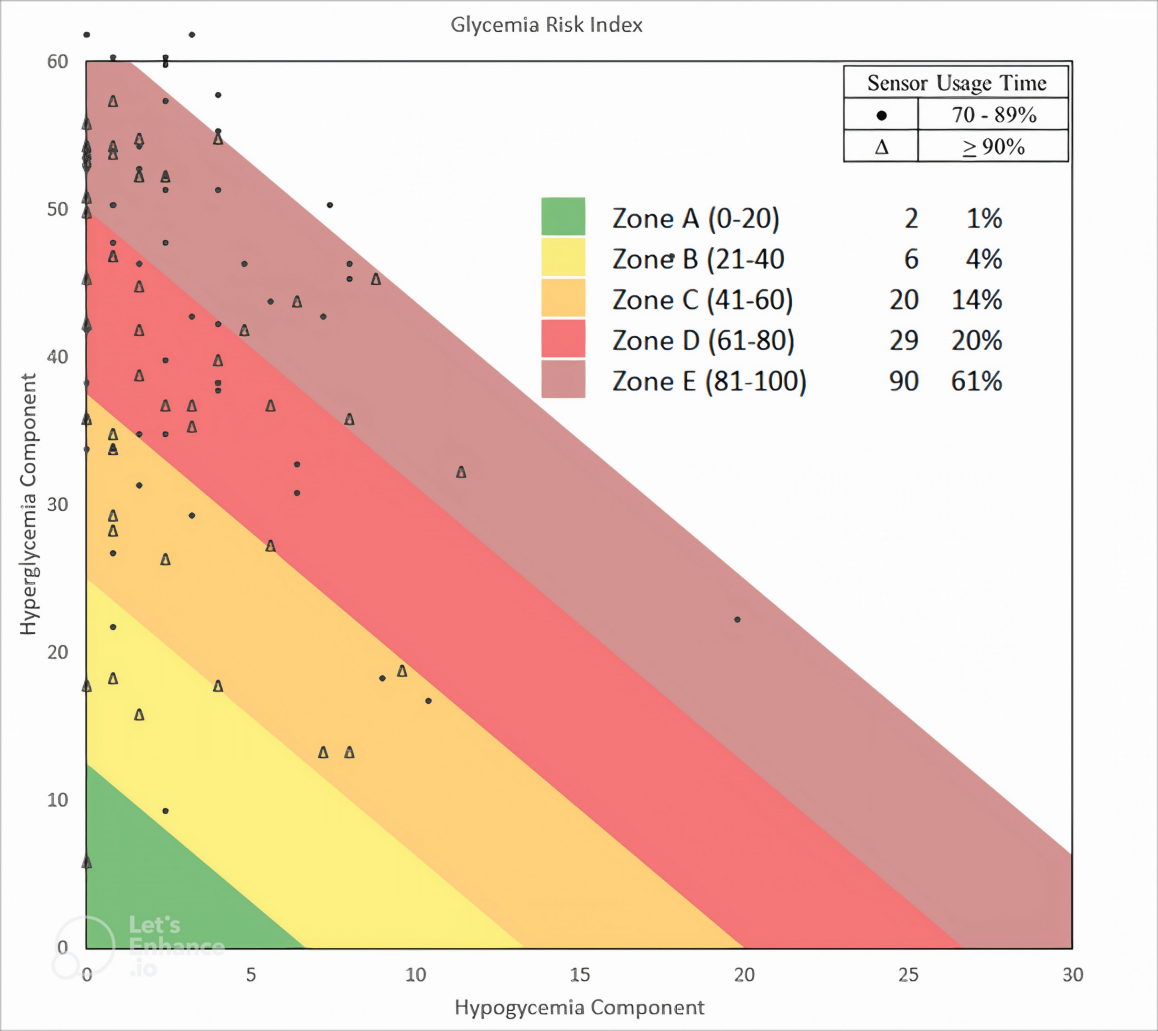
Glycemia Risk Index grid displaying hyperglycemia and hypoglycemia components according to sensor usage time, with distinct symbols representing two usage categories (n = 147).

The GRI grid graph in Figure 1 illustrates the distribution of hypoglycemia and hyperglycemia components across five GRI zones, categorized by two sensor usage times. The whole set of data had a higher hyperglycemia component. Subjects with sensor usage (>90%) were more in zones A, B, and C, and had a higher hypoglycemia component compared to subjects with sensor usage time (70%-89%) who were more in zone E and had a lower hypoglycemia component.

## Discussion

The extensive glycometric data generated by CGM systems have enabled the creation of new indices for assessing glycemic control in individuals with T1DM [13]. One such index is the GRI, a recently developed composite measure that quantifies "glycemic quality" by incorporating the severity of both hyperglycemia and hypoglycemia [11]. The GRI is easy to calculate and is based on the consensus of expert clinicians [14,15]. The present study aimed to explore how the GRI is associated with glycemic metrics obtained from the AGP, including the CGM sensor usage time among a population of pediatric patients with T1DM.

Our findings align with those of previous studies regarding glycemic parameters of centrality, where GRI showed a negative correlation with TIR, and positive correlations with ABG and GMI. The negative correlation between GRI and TIR in our study agrees with the results reported in the original article on the GRI [11]. It also aligns with findings from studies conducted among children [8,16] and adult populations [13,15,16]. Likewise, multiple studies among children [8,17] and adults [13,15] have demonstrated a strong positive correlation between GRI and ABG. Additionally, a study in children found a strong positive correlation between GRI and GMI [17], a finding also supported in a study among adults [13]. Similarly, our findings, which show a strong positive correlation between GRI and both extreme hyperglycemia (TAR2) and CHyper, are consistent with results from studies involving children [8,16] and adults [13]. These findings confirm the value of the GRI in assessing glycemic risk, as it shows a strong correlation with metric clusters that quantify hyperglycemia exposure. These clusters include indicators of treatment efficacy, such as TAR and ABG levels [18].

However, when examining glycemic parameters that reflect time spent below target values and moderate hyperglycemia, a different pattern emerged in this pediatric population. Specifically, both TAR1 (181-250 mg/dl), representing moderate hyperglycemia, and TBR1 (54-69 mg/dl), indicating moderate hypoglycemia, showed negative correlations with the GRI. This contrasts with previous studies in both pediatric [8] and adult populations [15,16], which reported positive or non-significant correlations for TAR1 [8,16] and TBR1 [8,13,15]. Contrary to expectations, in our study, as TAR1 and TBR1 increased, GRI values decreased. A similar inconsistency was observed in the correlation between TIR and hypoglycemia parameters. While a negative or no correlation would be expected, TIR showed a positive correlation with TBR1 (54-69 mg/dl) and TBR2 (<54 mg/dl). This may suggest that the observed decrease in the GRI is not necessarily due to increased time within the target glucose range. Rather, it reflects a shift from periods of extreme hyperglycemia and extreme hypoglycemia to more moderate deviations, specifically, from severe to moderate hyperglycemia and from severe to moderate hypoglycemia. This pattern may indicate that our population and their caretakers are using continuous glucose monitoring as a tool to reduce the risk of severe hyperglycemia by moderating their glucose levels, shifting from extreme to less severe elevations. While these moderate levels still exceed the recommended range, patients may view them as less urgent or dangerous compared to more extreme hyperglycemic episodes. This behavior could be attributed to the inherently uneven distribution of time spent outside the target glucose range (70-180 mg/dl), where elevations above the range are more common and often do not lead to acute complications that require immediate medical attention [4,9,19]. Further in-depth investigations that consider additional variables are needed to explain this inverse pattern among our population.

Our results also showed that GRI was not significantly correlated with TBR2, a finding consistent with other similar studies. This lack of correlation was attributed to the low incidence of hypoglycemia in the study population [15-17], a pattern that was observed in our study. Another possible explanation is that the GRI strongly correlates with hypoglycemia risk in individuals with HbA1c below 7%, while in those with HbA1c above 7%, it is more closely associated with hyperglycemia indices [20]. Although HbA1c was not measured in our study, the average GMI in this population was above 7%, which may explain the stronger correlation of GRI with hyperglycemia rather than hypoglycemia components.

The GRI’s sensitivity to hypoglycemia, due to its focus on extreme glycemic values rather than central measures, distinguishes it from metrics like HbA1c and TIR [13,21]. However, if the GRI shows weak or no significant correlations with CHypo or TBR in patient subgroups with a lower risk of hypoglycemia, its utility may be limited in these populations [13]. However, further studies in populations with varying levels of glycemia are needed before definitive conclusions can be drawn.

Our findings indicate that both GRI and CHyper improved with increased CGM sensor usage time. Achieving high sensor active time depends on correct insertion, proper activation, and regular monitoring of the CGM system [22]. These factors may reflect greater patient engagement and a stronger commitment to effective diabetes management [23]. Frequent glucose monitoring enhances individuals’ understanding of glycemic patterns, enabling timely adjustments and improved integration of CGM data into daily diabetes management [24-26], thus leading to improved glycemic metrics. Therefore, it is recommended that healthcare provider teams should prioritize effectively identifying, educating, supporting, and motivating patients to maximize the benefits of CGM use [24].

A key strength of this study is the use of real-world data, which enhances the applicability of the findings to routine clinical practice. However, limitations include the anonymization of data, which restricted access to important patient characteristics, such as treatment regimen, diet, and physical activity, thereby limiting the ability to assess their influence on GRI levels. The cross-sectional design further limits the ability to determine a temporal relationship between sensor use and GRI, introducing potential confounding. Additionally, unmeasured variables may have affected both sensor usage and GRI. The small sample size also reduces the precision of the findings and may limit their generalizability.

## Conclusions

In this population characterized by high levels of hyperglycemia and low levels of hypoglycemia, our findings align with previous studies on glycemic centrality parameters, including ABG, GMI, and TIR. This supports the validity of the GRI in assessing hyperglycemic exposure and serving as an indicator of treatment efficacy. Conversely, mild hyperglycemia (181-250 mg/dl) and mild hypoglycemia (54-69 mg/dL) unexpectedly showed negative correlations with GRI, warranting further investigation across populations with varying glycemic levels. Additionally, our results indicate that both GRI and CHyper improved with increased sensor usage time, suggesting that greater adherence to CGM may enhance overall glycemic control.
